# Preoperative vitamin D deficiency is a risk factor for postoperative hypocalcemia in patients undergoing total thyroidectomy: retrospective cohort study

**DOI:** 10.1590/1516-3180.2018.0336140319

**Published:** 2019-07-22

**Authors:** Genival Barbosa de Carvalho, Lina Restrepo Giraldo, Renan Bezerra Lira, Isabela Bergh Martins Macambira, Marcel Adalid Tapia, Hugo Fontan Kohler, Joel Arévalo Novoa, Luiz Paulo Kowalski

**Affiliations:** I MD, MSc. Attending Physician of Head and Neck Surgery, Department of Otorhinolaryngology, A.C. Camargo Cancer Center, and Head and Neck Surgery Sector, Department of Otorhinolaryngology, A.C. Camargo Cancer Center, São Paulo (SP), Brazil.; II MD. Master’s Student of Head and Neck Surgery, Department of Otorhinolaryngology, A.C. Camargo Cancer Center, São Paulo (SP), Brazil.; III MD, PhD. Attending Physician of Head and Neck Surgery, Department of Otorhinolaryngology, A.C. Camargo Cancer Center, São Palo (SP), Brazil.; IV MD. Resident of Head and Neck Surgery, Department of Otorhinolaryngology, A.C. Camargo Cancer Center, São Paulo (SP), Brazil.; V MD. Resident of Head and Neck Surgery, Department of Otorhinolaryngology, A.C. Camargo Cancer Center, São Paulo (SP), Brazil.; VI MD. Attending Physician of Head and Neck Surgery, Department of Otorhinolaryngology, A.C. Camargo Cancer Center, São Paulo (SP), Brazil.; VII MD. Resident of Head and Neck Surgery, Department of Otorhinolaryngology, A.C. Camargo Cancer Center, São Paulo (SP), Brazil.; VIII MD, PhD. Director of Head and Neck Surgery, Department of Otorhinolaryngology, A.C. Camargo Cancer Center, São Paulo (SP), Brazil.

**Keywords:** Hypoparathyroidism, Risk factors, Vitamin D, Thyroidectomy

## Abstract

**BACKGROUND:**

The relationship between preoperative vitamin D deficiency and postoperative hypocalcemia in cases of total thyroidectomy (TT) is a matter of controversy and may vary according to geographical scenarios and populations.

**OBJECTIVE:**

The objective here was to evaluate whether preoperative vitamin D deficiency was associated with postoperative symptomatic hypocalcemia in a population in South America.

**DESIGN AND SETTING:**

Retrospective cohort study on data from all patients undergoing total thyroidectomy, with or without central compartment lymph node dissection, from January 2014 to December 2017, at the A. C. Camargo Cancer Center.

**METHODS:**

Patients with benign thyroid disease (Graves’ disease, multinodular goiter or hyperthyroidism) or thyroid cancer who underwent primary total thyroidectomy with or without central compartment lymph node dissection were included. The exclusion criteria were simultaneous parathyroidectomy and conditions that could affect serum calcium levels. The data collected included patient demographics, thyroid pathology, extent of the surgical procedure and complications. Information on preoperative and postoperative calcium, parathyroid hormone (PTH) and vitamin D levels were retrieved from the medical records.

**RESULTS:**

1,347 patients were assessed and postoperative hypocalcemia was diagnosed in 284 patients (21%). The vitamin D levels were considered deficient in 243 patients (18%). Postoperative hypocalcemia was diagnosed in 357 patients (31.5%). Multivariate analysis showed that central compartment dissection and preoperative total calcium and deficient vitamin D levels were significant risk factors for postoperative hypocalcemia.

**CONCLUSION:**

Deficient preoperative vitamin D levels were a significant risk factor for postoperative hypocalcemia. Preoperative oral supplementation should be considered, to minimize this risk.

## INTRODUCTION

Techniques for thyroidectomy have evolved remarkably over the past 150 years. This is currently considered to be a very safe operation with favorable results when performed by experienced surgeons.^1^ Hypocalcemia as a result of hypoparathyroidism is the most common postoperative complication of thyroidectomy. Hypoparathyroidism is considered to be transient if recovery occurs within days, weeks or a few months; or permanent when calcium levels do not return to normal within six months after surgery.^2^^,^^3^ Transient hypoparathyroidism is seen in 0.3 to 49% of the patients undergoing thyroidectomy, whereas permanent hypoparathyroidism is less likely and has been reported in up to 13% of the cases.^4^^,^^5^^,^^6^^,^^7^^,^^8^

The established risk factors for hypoparathyroidism after total thyroidectomy are advanced age, female sex, size of the thyroid gland, substernal goiter, Graves’ disease, surgical technique (de-vascularization, excision or other inadvertent damage of the parathyroid glands), central compartment dissection, reoperation, less experienced surgeon and low 25-hydroxyvitamin D serum levels in the preoperative period.^9^^,^^10^^,^^11^^,^^12^^,^^13^^,^^14^^,^^15^^,^^16^^,^^17^^,^^18^

Most thyroid surgeons provide calcium supplementation based on postoperative calcium, parathyroid hormone (PTH) serum levels, or presence of symptoms, whereas others routinely prescribe calcium and vitamin D supplementation after thyroidectomy to prevent hypocalcemia symptoms.^12^^,^^13^^,^^19^ In a randomized study involving 143 patients undergoing total thyroidectomy, it was demonstrated that patients with PTH levels > 10 pg/ml on the first postoperative day could be safely discharged without routine calcium supplementation.^20^ The active form of vitamin D, i.e. calcitriol (1,25 dihydroxyvitamin D3), is the preferred option because of its potency and rapid onset of action.^21^

In the United States, the National Health and Nutrition Survey (NHANES), conducted from 2005 to 2006, showed that 41.6% of adults had levels of 25-hydroxyvitamin D (25-OHD) below 20 ng/ml.^22^ The prevalence of low vitamin D levels is also high globally.^23^^,^^24^^,^^25^ Low vitamin D levels (< 10 ng/ml [25 nmol/l]) are more common in South Asia and the Middle East than in other regions.^26^ Several risk factors, such as changes in milk intake, limited exposure to sunlight or use of sun protection, higher body mass index (BMI) and aging, compromise the absorption and metabolism of vitamin D.^24^ In several Brazilian regions, despite their geographical location in the tropics, there is high prevalence of hypovitaminosis D (up to 60%).^27^^,^^28^ Patients with prolonged vitamin D deficiency present reduced intestinal absorption of calcium and phosphorus.^29^

## OBJECTIVE

The relationship between preoperative vitamin D deficiency and postoperative hypocalcemia in patients who have undergone total thyroidectomy is not well defined. Thus, the objective of this study was to evaluate whether preoperative vitamin D deficiency was a risk factor for postoperative symptomatic hypocalcemia in patients in South America.

## METHODS

This was a retrospective study in which information collected from the databases of the Departments of Head and Neck Surgery and Otorhinolaryngology at the A.C. Camargo Cancer Center, Sao Paulo, Brazil, covering the period from January 2014 to December 2017, was analyzed. This study was approved by an Internal Review Board (Ethics Committee), under the number 2603/18, in September 2018.

The records of patients with benign thyroid disease (Graves’ disease, multinodular goiter or hyperthyroidism) or thyroid cancer who underwent primary total thyroidectomy, with or without association with central compartment lymph node dissection, were included. The exclusion criteria were presentation of simultaneous parathyroidectomy or conditions that could affect serum calcium levels, such as renal impairment, Paget’s disease, histiocytosis, hyperparathyroidism or use of thiazide diuretics or lithium.

The data collected included patient demographics, thyroid pathology, extent of the surgical procedure and complications. Information on preoperative and postoperative calcium, PTH and vitamin D levels were retrieved from the medical records. Data on preoperative vitamin D levels were only available for 395 (34.9%) of the patients.

Vitamin D levels were measured using the Elecsys total vitamin D electrochemiluminescence test, which was launched by Roche Diagnostic in 2012. This test is comparable to the liquid chromatography method performed in association with mass spectrometry (LC-MS/MS). This is an international reference method for measurement of vitamin D, in accordance with the Vitamin D External Quality Assessment Scheme (DEQAS), which is a worldwide reference program that has the objective of guaranteeing the reliability of vitamin D tests.^30^^,^^31^^,^^32^ Intact PTH was assayed using immunometric tests. Blood samples were collected and then refrigerated and subjected to rapid centrifugation. In the assays on PTH levels, the reference range was from 12 to 65 ­pg/­ml. The total calcium level was determined using an automated colorimetric method based on atomic absorption. Normality was taken to range from 8.4 to 10 mg/dl.

The surgeons tried to identify and preserve all parathyroid glands. In addition, every attempt was made to preserve the vascularization in the parathyroid glands. Ligature of the lower thyroid artery was usually performed at the level of the distal branches near the thyroid capsule. The parathyroid glands that were inadvertently resected or de-vascularized, or could not be preserved in situ, were cut into fragments and auto-transplanted into the ipsilateral sternocleidomastoid muscle using the technique described by Wells et al.^33^ Only patients with postoperative calcium levels below the normal value or who presented muscle cramps or tingling were treated with calcium and calcitriol replacement. The clinical sign of Chvostek was not used as a parameter because in most of the patients, no preoperative evaluation of this sign had been made, and patients with normal levels of calcium could also present it.

Statistical analysis was performed using Stata 14.2. Continuous variables were described in terms of the mean and standard deviation. Multiple imputation (MI) was used under the assumption that observations could be missing at random. Dependent and independent variables were used as imputation parameters for MI. Multivariate imputation by means of chained equations was used for data management, with 20 replications. Restricting the analysis to complete cases was deemed to be satisfactory if missingness was less than 5% and totally aleatory. Otherwise, it would rely on stronger *a priori* assumptions than random distribution.

The t test was used to compare means between two groups, whereas analysis of variance (ANOVA) was used when more than two groups were involved. Preoperative vitamin D levels were stratified accordingly and were classified as deficient (< 20 ng/ml), insufficient (between 21 and 30 ng/ml) or sufficient (> 31 ng/ml). Multivariate analysis was performed to identify factors predictive of hypocalcemia, using all the variables that were considered clinically significant. Because total calcium, vitamin D and PTH present interconnected metabolism, the interaction between these variables was tested.

A P-value of 0.05 was considered significant, and all tests were considered two-tailed. Variable selection and coefficient reduction were performed afterwards by means of the least absolute shrinkage and selection operator (LASSO). Using the selected variables, a nomogram was drawn to predict occurrences of postoperative hypocalcemia.

## RESULTS

The inclusion criteria were met by 1,347 cases. Most patients were women (1,070) and the age range was from 7 to 85 years (mean, 45.0 years; standard deviation, SD, 13.4 years). A total of 1,183 patients (89.9%) underwent total thyroidectomy alone. Central compartment dissection was performed in 164 patients (12.8%).

The mean preoperative serum total calcium level was 9.34 ­mg/­dl (range: 7.1 to 11.4 mg/dl), the PTH level was 36.7 pg/ml (range: 12.0 to 145.4 pg/ml) and the vitamin D level was 27.9 ­ng/­ml (range: 5.9 to 91.1 ng/ml). The five patients who had preoperative PTH levels above the reference value were not excluded from the analysis because they did not have the diagnosis of primary hyperparathyroidism, given that their serum total calcium levels were at the lower limit of normality. The PTH level was considered deficient in 243 patients (18%). A total of 390 patients (29%) had vitamin D insufficiency, while 714 patients (53%) had sufficient vitamin D. The mean preoperative PTH level was 41.0 ­pg/­ml (SD, 21.8) in patients with deficient vitamin D; 38.3 pg/ml (SD, 19.8) when the vitamin D level was classified as insufficient; and 31.9 pg/ml (SD, 12.8) in cases of sufficient vitamin D (Table 1). Comparing preoperative PTH levels according to categorical vitamin D level, the ANOVA test showed that there were lower PTH levels in patients with sufficient levels of vitamin D than in patients with deficient or insufficient levels (P < 0.001).

**Table 1.** Demographic characteristics of patients in relation to presence or absence of acute hypocalcemia

Variable		Acute hypocalcemia	No acute hypocalcemia	P-value
Age	Years	43.9 (12.71)	45.3 (13.62)	0.1219
Gender	Female	231 (17.15)	839 (62.29)	0.3051
	Male	52 (3.86)	225 (16.70)	
Malignancy	No	69 (5.12)	312 (23.16)	0.0695
	Yes	214 (15.89)	712 (52.86)	
CCD	No	217 (16.11)	966 (71.71)	< 0.001
	Yes	68 (5.05)	96 (7.13)	
Preoperative PTH		37.6 (20.14)	36.6 (17.49)	0.4540
Preoperative total Ca		9.3 (0.49)	9.3 (0.46)	0.9650
Preoperative vitamin D		26.1 (9.56)	28.5 (10.33)	0.0005

CCD = central compartment dissection; PTH = parathyroid hormone; Ca = calcium.

For the multiple imputation procedure, we initially tabulated our variables of interest, which showed 463 missing values for preoperative ionic calcium, 252 missing values for preoperative total calcium, 365 missing values for preoperative PTH, 48 missing values for postoperative PTH, 16 missing values for postoperative ionic calcium, 15 missing values for postoperative total calcium and 952 missing values for postoperative vitamin D. We then examined the pattern of missingness. A correlation matrix of potential auxiliary variables was created and, as no variable showed a correlation (r > 0.4), we created an imputation model using preoperative total calcium, preoperative PTH, age and postoperative hypocalcemia as the auxiliary variables.

Postoperative hypocalcemia was diagnosed in 357 patients (31.5%). Multivariate analysis showed that central compartment dissection and preoperative total calcium and deficient vitamin D levels were significant risk factors for postoperative hypocalcemia (Table 2). This model had an area under the curve (AUC) of 0.7226. The difference between preoperative and postoperative PTH was associated with preoperative vitamin D status: -22.7 (SD, 17.7) in cases of deficiency, -24.5 (SD, 19.9) in cases of insufficiency and -19.3 (SD, 13.1) in cases of sufficiency (P < 0.001).

**Table 2.** Multivariate analysis without interaction

Variable	Coefficient	95% CI	P-value
Neck dissection	1.195	0.751-1.639	< 0.001
Malignancy	0.223	0.0233-0.570	0.041
Gender	0.197	-0.220-0.614	0.354
Age	-0.004	-0.017-0.009	0.503
Preoperative total Ca	0.130	0.040-0.243	0.045
Preoperative PTH	0.006	-0.003-0.015	0.193
Preoperative vitamin D	-0.022	-0.040 - -0.004	0.015

CI = confidence interval; Ca = calcium; PTH = parathyroid hormone.

**Figure 1.**
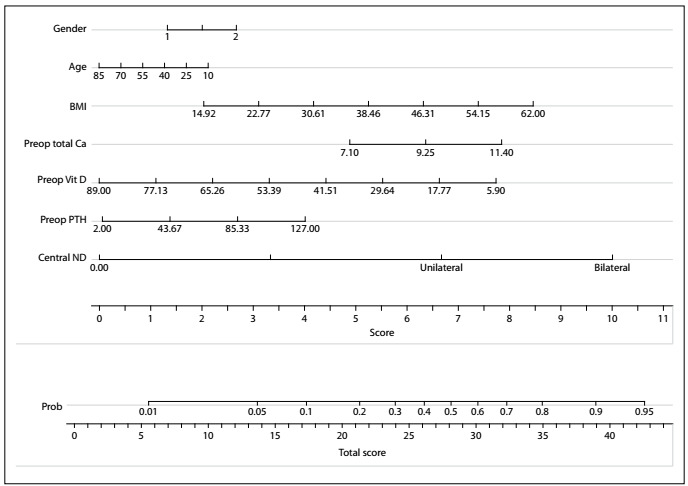
Nomogram with predictive factors for postoperative hypocalcemia in cases of total thyroidectomy.

Lastly, multivariate analysis with interaction terms alone showed that central compartment dissection and interaction between preoperative PTH and vitamin D were statistically significant (Table 3). This model had an AUC of 0.7430. After LASSO, the interaction terms of preoperative vitamin D and PTH, preoperative PTH, preoperative vitamin D, preoperative ionic calcium and neck dissection were selected. This model had an AUC of 0.6911. Based on these variables, a nomogram was designed (Figure 1). The objective of the nomogram was to provide an easy visual manner for estimating the preoperative risk of hypocalcemia in candidates for total thyroidectomy.

**Table 3.** Multivariate analysis with interaction terms

Variable	Coefficient	95% CI	P-value
CCD	1.207	0.710-1.703	< 0.001
Malignancy	0.111	-0.337-0.559	0.628
Gender	0.166	-0.296-0.629	0.481
Age	-0.003	-0.018-0.011	0.659
Preoperative total Ca	-0.393	-1.084-0.298	0.265
Preoperative PTH	0.037	-0.091-0.165	0.567
Preoperative vitamin D	-0.197	-0.430-0.036	0.098
PTH versus vitamin D	0.017	0.005-0.040	0.039
Total Ca versus vitamin D	0.001	-0.001-0.002	0.446
PTH versus total Ca	-0.005	-0.019-0.009	0.492

CI = confidence interval; CCD = central compartment dissection; Ca = calcium; PTH = parathyroid hormone.

## DISCUSSION

The present study demonstrated high incidence of vitamin D insufficiency (< 30 ng/ml) and deficiency (< 20 ng/ml) in a population from tropical areas and suggested that there was an association between vitamin D deficiency and the risk of postoperative hypocalcemia. The retrospective nature of the analysis and the fact that data on preoperative vitamin D values were available for only 34.9% of the patients were limitations of the study. However, when we compared the groups with and without vitamin D assays, we did not observe any differences, and our sample of more than 1,000 patients allowed adequate statistical analysis.

Activated vitamin D plays a central role in the regulation of calcium and PTH levels. Vitamin D increases serum calcium by directly increasing intestinal absorption and bone resorption while regulating PTH secretion through its effects on calcemia and through vitamin D receptors in the parathyroid glands.^34^^,^^35^ Because of these actions, the preoperative vitamin D level may have a profound impact on the perioperative kinetics of calcium and PTH after total thyroidectomy.^17^^,^^22^ Some clinical studies have found that low preoperative 25-hydroxyvitamin D (25-OHD) levels are a risk factor for postoperative hypocalcemia, whereas others have not found this association.^17^^,^^35^^,^^36^^,^^37^

Vitamin D deficiency is a cause of secondary hyperparathyroidism, and therefore the capacity of PTH levels in the postoperative period to act as a predictor of hypocalcemia probably depends on the preoperative vitamin D level. In Brazil, it was found that 62.1% of adolescents had vitamin D levels ranging from 11 to 30 ng/ml^28^ and that 85.6% of elderly patients had vitamin D levels below 20 ng/ml.^43^ In the present study, the prevalence of vitamin D deficiency was also high: 243 patients (18%) had vitamin D levels < 20 ng/ml and 390 patients (29%) between 20 and 30 ng/ml. On the other hand, the vitamin D levels that are deemed to be normal have varied over time and, moreover, the place where the study was conducted and the profile of the patients studied have also impacted the prevalence of vitamin D deficiency.^28^^,^^37^^,^^43^

Preoperative PTH levels are unreliable for predicting postoperative hypocalcemia because low levels of vitamin D lead to higher preoperative PTH levels due to secondary hyperparathyroidism.^17^^,^^41^ However, postoperative serum PTH levels in patients with vitamin D deficiency may be even higher than in those without vitamin D deficiency, despite the higher risk of hypocalcemia in the first group of patients.^17^ In patients with vitamin D deficiency, even minimal damage to the parathyroid glands caused by surgical manipulation may temporarily reduce PTH secretion and cause hypocalcemia, because these patients’ calcium regulation presents greater sensitivity to circulating serum PTH levels than that of individuals with normal levels of vitamin D. The role of vitamin D deficiency in causing parathyroid enlargement^42^ supports the idea that increased parathyroid gland activity compensates for low vitamin levels, thus making these patients more susceptible to hypocalcemia after thyroidectomy.

Total thyroidectomy is currently the standard surgical procedure for various thyroid diseases. It has the aim of reducing the incidence of recurrent disease and thus avoiding re-operations. Although it is considered to be a safe surgical procedure, postoperative hypoparathyroidism still affects a substantial number of patients. In addition, it has become a burden for the healthcare system because patients with hypocalcemia may require longer hospitalization, more biochemical studies, pharmacological treatments and more medical resources.^5^ Because of this, some authors have recommended routine supplementation of calcium and vitamin D to decrease the risk of biochemical and symptomatic hypocalcemia.^13^^,^^19^^,^^38^^,^^39^^,^^40^

Based on this study, we recommend that preoperative vitamin D correction should be undertaken. However, postoperative calcium and calcitriol replacement should be performed only when the patient develops symptoms of hypocalcemia or when calcium or PTH levels are below normal.

## CONCLUSIONS

Deficient preoperative vitamin D levels are a significant risk factor for postoperative hypocalcemia, as are also central compartment neck dissection and total preoperative calcium level. Therefore, preoperative oral supplementation of vitamin D should be considered, to minimize this risk.
